# Curcumin as an Alternative Epigenetic Modulator: Mechanism of Action and Potential Effects

**DOI:** 10.3389/fgene.2019.00514

**Published:** 2019-06-04

**Authors:** Faiz-ul Hassan, Muhammad Saif-ur Rehman, Muhammad Sajjad Khan, Muhammad Amjad Ali, Aroosa Javed, Ayesha Nawaz, Chengjian Yang

**Affiliations:** ^1^Key Laboratory of Buffalo Genetics, Breeding and Reproduction Technology, Ministry of Agriculture and Guangxi Buffalo Research Institute, Chinese Academy of Agricultural Sciences, Nanning, China; ^2^Institute of Animal and Dairy Sciences, University of Agriculture Faisalabad, Faisalabad, Pakistan; ^3^Faculty of Veterinary Sciences, Bahauddin Zakariya University, Multan, Pakistan; ^4^Department of Zoology, Wildlife and Fisheries, University of Agriculture Faisalabad, Faisalabad, Pakistan

**Keywords:** curcumin, cancer, epigenetic modulation, alternative treatment, angiogenesis

## Abstract

Curcumin (a polyphenolic compound in turmeric) is famous for its potent anti-inflammatory, anti-oxidant, and anti-cancer properties, and has a great potential to act as an epigenetic modulator. The epigenetic regulatory roles of curcumin include the inhibition of DNA methyltransferases (DNMTs), regulation of histone modifications via the regulation of histone acetyltransferases (HATs) and histone deacetylases (HDACs), regulation of microRNAs (miRNA), action as a DNA binding agent and interaction with transcription factors. These mechanisms are interconnected and play a vital role in tumor progression. The recent research has demonstrated the role of epigenetic inactivation of pivotal genes that regulate human pathologies such as cancers. Epigenetics helps to understand the mechanism of chemoprevention of cancer through different therapeutic agents. In this regard, dietary phytochemicals, such as curcumin, have emerged as a potential source to reverse epigenetic modifications and efficiently regulate the expression of genes and molecular targets that are involved in the promotion of tumorigenesis. The curcumin may also act as an epigenetic regulator in neurological disorders, inflammation, and diabetes. Moreover, curcumin can induce the modifications of histones (acetylation/deacetylation), which are among the most important epigenetic changes responsible for altered expression of genes leading to modulating the risks of cancers. Curcumin is an effective medicinal agent, as it regulates several important molecular signaling pathways that modulate survival, govern anti-oxidative properties like nuclear factor E2-related factor 2 (Nrf2) and inflammation pathways, e.g., nuclear factor kappa B (NF-κB). Curcumin is a potent proteasome inhibitor that increases p-53 level and induces apoptosis through caspase activation. Moreover, the disruption of 26S proteasome activity induced by curcumin through inhibiting DYRK2 in different cancerous cells resulting in the inhibition of cell proliferation opens up a new horizon for using curcumin as a potential preventive and treatment approach in proteasome-linked cancers. This review presents a brief summary of knowledge about the mechanism of epigenetic changes induced by curcumin and the potential effects of curcumin such as anti-oxidant activity, enhancement of wound healing, modulation of angiogenesis and its interaction with inflammatory cytokines. The development of curcumin as a clinical molecule for successful chemo-prevention and alternate therapeutic approach needs further mechanistic insights.

## Introduction

Curcumin, scientifically known as diferuloylmethane, is a yellow polyphenol and the active component of the perennial herb *Curcuma longa*, usually known as turmeric (Aggarwal and Sung, 2009). It contains 80% curcuminoid complex, 17% dimethoxy-curcumin, and 3% bisdemethoxy-curcumin (Lao et al., 2006). Curcumin is well known for its potent anticancer activities which have been systematically examined through elucidation of a number of potential mechanisms of actions. Even though, pharmacokinetic studies revealed that curcumin is found in quite lesser plasma concentration in human beings as compared to *in vitro*, yet many preclinical investigations have confirmed the anticancer activities of curcumin (Cheng et al., 2001; Sharma et al., 2004). Curcumin can provoke the apoptosis but slow down the proliferation of cancer cell lines (Huminiecki et al., 2017). The active biological activity of curcumin at slightly low concentrations in humans might be due to epigenetic modulation of different pathways. Epigenetics leads to heritable alterations in the expression of genes while maintaining their coding DNA sequences. Moreover, it provides an effective approach to discriminately inactivate or activate the expression of genes by endogenous and exogenous substances (Davis and Ross, 2007). Mechanisms of epigenetics include changes in DNA methylation, histone modification, and alteration in the expression of miRNAs (Yoo and Jones, 2006; Winter et al., 2009). Natural complexes like resveratrol, epigallocatechin gallate (EGCG) and curcumin have been known to induce epigenetic changes that may enhance the sensitivity of cancerous cells to usual chemo-therapeutic agents and consequently suppress tumor growth (Li et al., 2010).

Curcumin is an effective therapeutic compound, because it controls many important pathways of molecular signaling which in turn modulate the survival and pathways that govern anti-oxidative factors (such as nuclear factor E2-related factor 2, Nrf2) and inflammatory responses like nuclear factor kappa B (Hatcher et al., 2008). Other than its role in controlling Nrf2 in various kinds of cancers, curcumin harmonizes Nrf2 expression in various human ailments such as neurocognitive disorders, diabetes, and renal disorders. Curcumin is well known as an anti-inflammatory mediator because it controls the anti-inflammatory reaction by decreasing the activities of cyclooxygenase (COX-2) and inducible nitric oxide synthase (iNOS) via withholding the transcription of nuclear factor kappa B (NF-κB), ultimately leading to the cessation of tumorigenesis (Surh et al., 2001). Curcumin also reduces the expression of genes being regulated by NF-κB, including 5-lipoxygenase (5-LOX), tumor necrosis factor (TNF), adhesion molecules, interleukins (IL-1, IL-6, IL-8), chemokine receptor type 4 (CXCR-4), and C-reactive protein (Rao, 2007; Skommer et al., 2007).

Studies conducted on small intestine and liver of mice have shown that curcumin can suppress or induce the expression of many genes associated with the regulation of cell cycle, apoptosis, cell adhesion, phosphatases, and kinases. The changes in several phase II antioxidant/detoxification enzymes that are controlled by Nrf2 indicate the possible roles of curcumin and Nrf2 in lowering the risk of cancer (Shen et al., 2006). A recent study in mice revealed that curcumin is a neuro-protectant with respect to hemin-induced damage among foremost cultures of cerebellar granule neurons. These neuroprotective effects were mediated through glutathione (GSH) synthesis or inhibition of the heme oxygenase system by means of buthionine sulfoximine and tin mesoporphyrin, respectively. Moreover, the activities of glutathione *S*-transferase, glutathione reductase and superoxide dismutase were enhanced by 2.3-, 1.4-, and 5.2-fold, respectively, after a 24-h incubation with curcumin. These findings suggested that an antioxidant response and Nrf2 activation may have contributed significantly to the protective effect of curcumin against neuronal death induced by hemin (González-Reyes et al., 2013).

Curcumin is a unique molecule with diverse biological activities, including its beneficial effects on diabetes, particularly regarding insulin sensitivity. Studies have reported that curcumin reduced glucose intolerance without influencing weight gain by inducing the nuclear translocation of Nrf2 along with its downstream target of heme oxygenase-1, which was decreased by a high-fat diet (He et al., 2012). Nrf2 is recognized as a major stress responsive factor that ameliorates adverse effects of different stressors like xenobiotics, inflammation, excessive metabolites, and misfolded proteins. Investigations on the molecular mechanisms responsible for the apparent anti-oxidant potential of curcumin proved the ability of curcumin to protect nerve cells against ischemic abrasion by involving the Akt/Nrf2 pathway (Wu et al., 2013). Investigation of ischemia-reperfusion injury in Nrf2−/− mice revealed noticeably poorer vascular permeability, kidney function, and survival in relation to wild-type mice. Diabetic nephropathy model induced by streptozotocin (STZ) revealed that Nrf2−/− mice had severely injured kidney and damaged DNA in comparison with normal mice. Treatment by cyclosporine A resulted in relatively higher interstitial fibrosis and kidney damage in Nrf2−/− mice (Liu M. et al., 2009; Jiang et al., 2010; Shin et al., 2010). It was further noticed that the provision of curcumin (100 mg/kg) greatly reduced the infiltration of macrophages into kidney and the expression of pro-inflammatory cytokines, including TNF-α and IL-1β accompanied with the inhibition of NF-κB in animals suffering from STZ diabetes (Soetikno et al., 2011). Moreover, curcumin also ameliorated adverse effects of arsenic induced hepatotoxicity and oxidative damage by reducing elevated serum levels of liver enzymes (AST and ALT) and the inflation of hepatic malondialdehyde (MDA). It also decreased hepatic and blood GSH levels through the activation of Nrf2 (Gao et al., 2013). Additionally, curcumin also catalyzes the expression of genes that transcribe anti-oxidant enzymes (Rogers et al., 2012; Trujillo et al., 2013). Above mentioned diverse array of biological functions mediated by curcumin provides a glimpse of its therapeutic potential in different human diseases.

## Epigenetic Modulation by Curcumin

Different studies have clearly described the potent role of curcumin as an epigenetic modulator. Most significant activities of curcumin are summarized in following aspects.

### Histone Deacetylation/Acetylation

Histone acetylation and deacetylation are two major histone modifications which are considered as significant epigenetic changes to alter the expression of genes. Their imbalance can lead to the risk of cancer (Gibbons, 2005). Histone deacetylases (HDACs) are the enzymes that interact with DNA by means of multiprotein compounds, including co-activators and co-repressors. HDACs eliminate the acetyl group from histone proteins which are associated with gene silencing while histone acetyltransferases (HATs) cause acetylation relevant to gene transcription. The balance between acetylation and deacetylation is important for the regulation of gene function. Irregular activities of HDACs and HATs have been associated with the onset of cancer. Around 18 HDACs have been identified which are grouped into four classes based on their similarities with yeast deacetylases (Xu et al., 2007).

The inhibition of HDACs by different substances, is being considered as a cancer therapeutic approach owing to their potential for regulating many cellular activities (Ceccacci and Minucci, 2016; Li and Seto, 2016). Potential effects of curcumin on the activities of HDACs/HATs proved that curcumin is the most potent inhibitor of HDACs as presented in Table 1 (Bora-Tatar et al., 2009). Curcumin has also been proven to be more effective when compared to sodium butyrate and valproic acid, which are considered as popular inhibitors of HDACs. Furthermore, the use of curcumin significantly reduced the levels of class I HDACs, leading to an increased level of acetylation (Liu et al., 2005; Chen et al., 2007, 2013). Curcumin has shown inhibition of ∼50% HDAC activity at a very high concentration of 500 μM in HeLa nuclear extracts (with IC50 value of 115 μM). It has also been reported that curcumin induced global inhibition of HDAC activity and reduced HDAC8 isoform activity while increasing the expression of suppressors of cytokine signaling, SOCS1 and SOCS3 in the leukemic cell (Chen et al., 2013). Moreover, curcumin exhibited increased acetylation of histone H4 by decreasing levels of HDAC1, 3 and 8 in the Raji cells (Liu et al., 2005). Direct inhibition of HDAC4’s transcription leading to reduced overall HDAC activity, has been revealed by treatment of the medulloblastoma cells with curcumin (Lee et al., 2011). These findings proved that curcumin is a potent inhibitor of HDAC activity with free binding energy and inhibition constant (for HDAC8) comparable to trichostatin A and vorinostat (Bora-Tatar et al., 2009). Conversely, the restoration of HDAC2 level in affected lungs in chronic respiratory disorders has also been observed by the use of curcumin. These contradictive findings revealed that the effect of curcumin on HDAC activity is variable and probably cell line specific (Meja et al., 2008).

**TABLE 1:** Epigenetic mechanisms modulated through Curcumin.

**Molecular mechanisms**	**Biological activities of curcumin**	**References**
DNA methylation	1. Covalently obstructs the catalytic thiolate of C1226 of DNA methyltransferase I	Liu Z. et al.,2009
	2. Suppress methyltransferase M.SssI at an IC50 of 30 nM	
	3. Provokes global genomic DNA hypo-methylation	
	4. WIF-I promoter hypomethylation; demethylation of NrF2 promoter	Liu et al., 2011; Khor et al.,2011
	5. Reduces methylation in promoter of Neurog1	Shu et al.,2011
	6. DNA hypomethylation	Jha et al., 2010; Abusnina et al., 2011; Du et al.,2012
	7. Demethylation of RAR2 gene	Parashar et al.,2012
	8. Impedes DNMT3B	Zheng et al., 2014; Jiang et al.,2015
Histone acetylation (acetyltransferases)	1. CBP/HAT inhibition	Balasubramanyam et al.,2004
	2. HAT inhibition	Kang et al.,2006
	3. Enhances p300 degradation and inhibits histone hyperacetylation	Marcu et al.,2006
	4. Down-regulates p300	Ryu et al., 2005; Chen et al.,2007
	5. H3 and H4 activation along with acetylation of p53	Chen et al., 2004; Shankar and Srivastava,2007
	6. Suppress GCN5 linked with hypo-acetylation of histone H3	Cui et al.,2007
	7. Direct inhibition of p300	Morimoto et al.,2008
	8. HAT activity suppression, hypoacetylation of p65 isoform of NF-κB	Yun et al.,2011
Histone deacetylation	1. Functions as an HDAC2 activator	Barnes,2009
	2. Enhances HDAC2 protein expression	Meja et al.,2008
	3. Functions as an HDAC8 inhibitor	Bora-Tatar et al., 2009; Chen et al.,2013
	4. Down-regulates HDAC1, 3, and 8	Chen et al., 2007; Hu et al.,2009
	5. HDAC3 and HDAC4 Inhibition and decrease in total HDAC activity	Lee et al., 2011; Shu et al.,2011
miRNAs	1. Upregulates miR-15a and 16	Yang et al.,2010
	2. Upregulates tumor suppressive miRNA panel (*let-7a,b,c,d*, *miR-26a*, *miR-101*, *miR-146a*, and *miR-200b,c*)	Bao et al.,2012
	3. Downregulates miR 125-5p	Gao et al.,2014
	4. Downregulates miR-19a and 19b	Li et al.,2014
	5. Upregulates miR-181b	Kronski et al.,2014
	6. Upregulates miR-9	Yang et al., 2013; Zhao et al.,2014
	7. Upregulates miR-145	Yu et al.,2013
	8. Downregulates miR-27a while upregulates miR-34a	Toden et al.,2015
	9. Downregulates miR-130a	Kara et al.,2015

Potential therapeutic role of tumor suppressor gene p53 is well established in human cancer pathogenesis, as up-regulation of p53 could inhibit cellular proliferation through inducing cell cycle arrest and apoptosis in cancer cells (Kruse and Gu, 2009; Gong et al., 2018; Su et al., 2018). P53 is an important tumor suppression protein with dual activity as transcription activator and repressor being associated with cell proliferation, DNA repair and cell malignancy. Mutated or non-functional p-53 have been found in more than 50% human cancers leading to apoptotic resistance and continued proliferation (Kim and An, 2016). Pharmacological re-activation of p53 in treating cancers is envisioned as an effective therapeutic strategy as restoration of p53 results in apoptosis in lymphoma while suspends cell growth and senescence in sarcoma (Ventura et al., 2007). Activation of p53 by acetylation leads to its binding with DNA that ultimately mediates transcription of downstream targets (like GADD45 and p21) to arrest cell cycle and induce apoptosis (Gu and Roeder, 1997). Curcumin has shown acetylation of p53 leading to activation of p53 signaling pathway (Fu et al., 2018). The acetylated p53 recruits HATs like p300, CBP, etc., for further histone (H3 and H4) acetylation at the acetylated p53 binding sites leading to enhanced transcription of specific genes. Moreover, acetylation of p53 is also responsible for sustaining the combination of HATs and p53 along with maintenance of activity of p53 (Barlev et al., 2001; Labuschagne et al., 2018).

It has also been reported that HDAC1 induces deacetylation of p53 leading to its degradation (Ito et al., 2002). Curcumin has shown inhibition of HDAC1 leading to upregulation of the acetylated H3 and p53, that ultimately mediates tumor suppression and apoptosis (Balasubramanyam et al., 2004). In addition to acetylation, curcumin has also shown cytoplasmic activation, nuclear translocation and phosphorylation of p53 protein (on serine 15 moiety) which increases its level in cancer cells (Liontas and Yeger, 2004; Pan et al., 2008). P53 protein plays a vital role in cellular adaptive response to environmental stress. Positive correlation between p53 levels and treatment response in cancer therapy has been observed while decrease in p53 levels showed chemo-resistance in cancer cells. Moreover, mutated p53 have exhibited oncogenic properties and negative chemo-resistance effects as tumors with mutated p53 showed poor response to cancer therapy in lung and prostate cancers (Robles and Harris, 2009). Curcumin has also shown induction of ROS synthesis that increases p53 levels and its downstream proteins p21 and Bax (Thayyullathil et al., 2008). Moreover, curcumin has shown apoptosis independently of p53 particularly in the cells without functional p53 protein by downregulating Bcl-2 and p38 MAPK (Watson et al., 2010). Alternatively, curcumin can activate PPARγ which in turn transactivates p53 leading to mediation of cell senescence (Jin et al., 2016). Curcumin alone can induce the expression of p53 gene (Trp53) restoring the level as well as function of p53 (Das and Vinayak, 2015). It is reported that level of p53 in cells is controlled through many routes but major route is p53-murine double minute 2 (MDM2) pathway through an auto-regulatory feedback loop. Curcumin has shown downregulation of MDM2 leading to upregulation of the expression of p53 and Bax in multiple myeloma cancerous cells (Li et al., 2015). In conclusion, it is revealed that curcumin is effective for activation and restoration of p53 levels especially in the cells with an abnormal p53 expression or function. For example, curcumin has shown cell cycle arrest (at G2 phase) in p53 deficient breast cancer cells.

Abnormal activity of both HDACs and HATs is associated with the cancer pathogenesis, as a balance between histone acetylation and deacetylation is required for normal cellular physiology. Molecules like curcumin that modulate both HDACs and HATs to restore this balance possess significant anti-tumor potential. Curcumin has shown inhibition of the activity of certain isoforms of HAT and considered as a first natural selective HAT inhibitor (Devipriya and Kumaradhas, 2013). Recently, an *in vitro* study revealed partial inhibition of acetylation of histone H3K9 along with reversal of upregulation of the caspase activity (Caspase-3 and 8) and downregulation of Bcl-2 by curcumin in alcohol induced apoptosis in cardiac cells (Yan et al., 2017). Curcumin has also shown inhibition of acetylation of non-histone proteins like HIV-Tat protein leading to obstruct viral proliferation, exhibiting its potential as an adjuvant in HIV therapy (Morimoto et al., 2008).

Curcumin has been found to be specifically linked with the suppression of acetylation (of histone and p53) through inhibiting p300/CREB binding protein HAT activity from chromatin but not a DNA template. Curcumin is specific inhibitor of p300/CBP family with no effects on other HATs like PCAF/GCN5 (Balasubramanyam et al., 2004; Marcu et al., 2006). This selective inhibitory effect of curcumin is mediated by its Michael reaction acceptor function and makes curcumin a better pharmacological molecule with an anticancer therapeutic potential. The inhibition of p300/CBP can lead to degradation of p53 but it was not observed to a greater extent because p53 can be simultaneously acetylated by other HATs (not inhibited by curcumin) which restore its acetylation status in physiological range even after curcumin treatment. Moreover, curcumin triggered caspase-3 and poly (ADP-ribose) polymerase-mediated apoptosis among glioma cells by histone hypo-acetylation (Kang et al., 2006). The acetylation/deacetylation of molecular chaperones, transcription factors, cytoskeletal, and effector proteins is being focused as an important epigenetic regulatory approach (Glozak et al., 2005). NF-κB is a pro-inflammatory transcription agent which undergoes acetylation prior to the activation of hundreds of genes concerned with varied cellular processes (Gupta et al., 2010). NF-κB is acetylated at numerous lysine residues by means of p300/CBP acetyltransferases. Curcumin has also been reported to suppress the acetylation of RelA mediated by p300 (Chen et al., 2001).

Similarly, curcumin greatly decreased the expression of acetylated CBP/p300 HAT, leading to the inhibition of NF-κB binding (Yun et al., 2010). Additionally, curcumin suppressed the acetylation resulting from hypertrophy and binding of GATA4 that is a hypertrophy-responsive transcription agent in rat cardiomyocytes. It signifies that the suppression of activity of p300 HAT by curcumin might serve as a potential therapeutic intervention for heart failure in humans (Morimoto et al., 2008). Lastly, curcumin also incited the re-controlling fates of neural stem cell through the reduction of intensities of acetylation of H3 and H4 histone proteins (Kang et al., 2006).

Curcumin can modify both HATs and HDACs through similar mechanisms. For instance, oxidative pressure can stimulate NF-κB by the activation of natural HAT activity which results in the expression of pro-inflammatory mediators; on the other hand, it may suppress the activity of HDACs (Rahman et al., 2004). Consequently, curcumin may control acetylation and deacetylation by modulating oxidative stress. Curcumin modifies N-terminal tail regions of histones (H3, H4, and H2A) that ultimately affect many cell signaling pathways, leading to altered expression of many genes (Fu and Kurzrock, 2010; Azad et al., 2013). This modification can influence a diverse array of cellular processes like transcription, cell cycle, differentiation, DNA repair and recombination, etc. (Strahl and Allis, 2000; Duncan et al., 2008; Scully, 2010). Recently, treatment with curcumin revealed the inhibition of p300 HAT activity, leading to a decreased acetylation of pro-nociceptive proteins (BDNF and Cox-2) in neuropathic pain in mice model (Zhu et al., 2014). Moreover, curcumin has also been shown to modulate DNA damage response pathways by inhibiting HAT activities (Ogiwara et al., 2013). Histone modifications mediated by curcumin are not well defined except its function in acetylation, and thus require further investigations to provide insights into its potential mechanism of action.

### DNA Methylation

Methylation has a vital role in managing normal biological activities in living systems (Esteller, 2007). Methylation of DNA is a type of transmissible change in the DNA that does not alter coding nucleotide sequence, however, it can directly suppress the expression of a gene (Das and Singal, 2004). Both hypo-methylation and hyper-methylation of DNA have been observed within cancer cells. Hypo-methylation can assist the expression of pro-metastatic genes and quiescent proto-oncogenes, and then enhance the progression of tumor. Localized hyper-methylation at particular CpG islands in promoter sections of particular genes (e.g., genes linked with tumor suppression) may lead to the silencing of transcription and a failure to control tumor development (Ehrlich, 2009).

S-adenosyl-methionine functions as a donor of methyl group for DNA methylation in the presence of DNA methyl-transferases, including DNMT1, DNMT3a, and DNMT3b, to produce 5-methylcytosine (Herman and Baylin, 2003). Studies exploring the influence of curcumin on DNA methylation are summarized in Table 1. Curcumin has been shown to inhibit the activities of DNMTs and then significantly modify the pattern of DNA methylation in different tumor cells (Liu Z. et al., 2009; Jha et al., 2010; Kuck et al., 2010; Link et al., 2013). Molecular docking studies revealed covalent blockage of catalytic thiolate of DNMT1 by curcumin, leading to hypo-methylation (Liu Z. et al., 2009). Curcumin induced reversal of DNA methylation in Leukemia cells, an action comparable to decitabine (a potent hypo-methylating agent) in global DNA methylation studies. Moreover, it also induced demethylation and expression of Neurog1 in LNCaP prostate cancer cells (Shu et al., 2011). The effect of curcumin on global hypo-methylation of DNA was not observed as it did not modify the methylation pattern of long interspersed nuclear elements-1 (LINE-1) in human colon cancer cells. However, it reduced the methylation of genes related to NF-κB pathway. Interestingly, it has been observed that hypo-methylating activity of curcumin was dependent on the density of methylation owing to selective demethylation of partially methylated CpG sites other than fully methylated genes (Link et al., 2013). Moreover, treatment with curcumin exhibited reactivation of silenced tumor suppressive genes by inducing demethylation of promoters of these genes (e.g., RARβ2 in human cervical cancer cell lines and *p15^INK4B^* in acute myeloid leukemia), leading to a remarkable tumor suppression (Jha et al., 2010; Yu et al., 2013). Similarly, it also induced the reversal of methylation of Nrf2 promoter in prostate cancer cells (Khor et al., 2011).

Contrarily, some studies reported no demethylation activity by curcumin as no significant global DNA hypo-methylation was observed in both leukemia and colorectal cancer following the treatments with curcumin (Medina-Franco et al., 2010; Link et al., 2013). Later on, these contradictive findings were confirmed by Hassan et al. (2015) based on the analysis of global DNA methylation by DNA pyrosequencing. They reported that both curcumin and its structural analog dimethoxycurcumin (DMC) did not reveal any significant hypo-methylation activity even at very high concentrations. Surprisingly, they observed the induction of expression of promoter-methylated genes by DMC without reversing DNA methylation. Previous studies also supported these findings as the induction of expression in respective genes has been observed in methylated promoters (Pruitt et al., 2006; Raynal et al., 2012).

FDA has approved two hypo-methylating agents namely 5-azacytidine and decitabine for curing myelodysplastic syndrome (MDS). Both of these have potential to make cancer cells more sensitive toward chemotherapeutic agents. It would be valuable to discover how the variable hypo-methylation induced by curcumin can trigger chemo-sensitization in cancers. Importantly, an initial trial using docetaxel after treatment with curcumin in patients of metastatic breast cancer ensured temporary recoveries in five patients while disease remained constant in three out of eight patients (Bayet-Robert et al., 2010). Such unpredicted responses may be due to release of these two agents in a sequential way. The sequential release of both of these hypo-methylating agents maximized epigenetic activity of curcumin for the treatment of cancer. Additionally, use of curcumin in various cancer models has proved that it could be utilized as a chemosensitizer for treatment of cancer.

### miRNA Expression

miRNAs are described as tiny non-coding regulatory RNAs consisting of 17–25 nucleotides (Croce, 2009). They reduce the rate of translation and/or enhance the destruction of mRNAs when they are expressed abnormally. They also play significant roles in cell differentiation, cell cycle, apoptosis, metastasis, angiogenesis, invasion, and development of tumors (Negrini et al., 2007). Further, 50 miRNA genes have been recognized in humans and it is hypothesized that about 500 human miRNA genes have yet to be discovered. The actual functional role of many miRNAs is still unknown in mammals (Bentwich et al., 2005). However, it is considered that in humans about 30% of the genome could be regulated by miRNAs (Bartel, 2004).

Instability of miRNA expression, processing of precursors of miRNA, changes in miRNA sequence and its target mRNA, may impart negative influences on cellular activities and is considered to be linked with cancer (Davis and Ross, 2008). Formation of cancerous stem cells along with typically drug resistant epithelial-mesenchymal transition (EMT) phenotype of cancerous cell lines is controlled by a few miRNAs (Li et al., 2010). Moreover, genes responsible mainly for signaling pathways including Akt, NF-κB, and MAPK are regulated by curcumin (Mukhopadhyay et al., 2001; Sarkar and Li, 2004). Similarly, miRNAs are also capable of regulating these cellular signaling pathways. Therefore, functional modulation of miRNA is considered as a rational therapeutic approach for the treatment of different types of cancers.

Curcumin has been reported to modulate the expression of miRNA within human pancreatic cancer cells. Based on curcumin incubation for a period of 72 h, a considerable up-regulation was noticed in 11 miRNAs while 18 miRNAs were down-regulated, of which miRNA-22 was most up-regulated while miRNA-199a was most down-regulated. Curcumin induced up-regulation of miRNA-22 inhibited the expression of target genes, including estrogen receptor 1 and Sp1 (Sun et al., 2008). These findings suggested that the regulation of particular miRNAs through curcumin can suppress the growth of pancreatic cancerous cells. Moreover, programmed cell death among A549/DDP multidrug-resistant human lung adenocarcinomic cells may be prompted through curcumin by miRNA-based signaling pathway. Curcumin mainly showed the down-regulation of miRNA-186 expression in these cells (Zhang et al., 2010).

Epigenetic manipulation of miRNAs by curcumin has been observed in pancreatic cancer cells in a study with identification of around 50 candidate genes targeted by miRNA-22 (Sun et al., 2008). Additionally, curcumin has also shown to induce gemcitabine sensitivity in pancreatic cancer cells by altering the expression of miR-21 and miR-200 (Ali et al., 2010). The miRNA-200 has potential to suppress EMT, the introductory step of metastasis, through regulation of the epithelial cellular network by directly targeting transcriptional inhibitors of E-cadherin, ZEB1, and ZEB2 (Korpal et al., 2008). Thus, it would be a significant therapeutic approach to target particular miRNAs for cancer treatment. It could be done by eradicating drug tolerant EMT-type cells or cancer stem cells.

In contrast, miRNA-21 is overexpressed in various tumors and it facilitates invasion, metastasis, and cancer. Therefore, it is considered as an oncomiR. Moreover, an increased concentration of miRNA-21 has been observed in chemotherapy resistant colorectal cancer patients (Yu et al., 2013). Treatment with curcumin reduces the activity of miRNA-21 promoter. It also decreases the expression of miRNA-21 in primary tumors by suppressing the binding of AP-1 to the promoter while initiating the expression of the tumor inhibitors such as Pdcd4 which is a target of miRNA-21 (Mudduluru et al., 2011).

Treatment with curcumin revealed the up-regulation of miRNA-203 expression but a down-regulation of its target genes (Akt2 and Src), ultimately leading to a reduced proliferation but an increased apoptosis in bladder cancer cells (Saini et al., 2011). These effects were mediated by the hypo-methylation of the promoter region of miRNA-203 induced by curcumin. Similarly, curcumin up-regulated the expression of miRNA-15a and miRNA-16 while suppressing anti-apoptotic protein (Bcl-2) in breast cancer cells (Yang et al., 2010). The up-regulation of tumor suppressive miRNAs (*let-7a,b,c,d*, *miR-26a*, *miR-101*, *miR-146a*, and *miR-200b, c*) has also been mediated by curcumin in pancreatic carcinomas (Bao et al., 2012). A higher expression of miRNA-130a has been associated with chemo-resistance in patients with colon cancers, resulting in poor clinical outcome (Kara et al., 2015). Recently, it is reported that curcumin mediated a down-regulation of miR-130a, leading to the activation of Wnt/β-Catenin in colon cancer (Dou et al., 2017). Similarly, the down-regulation of miR-27a (oncogenic) but the upregulation of miR-34a were induced by curcumin accompanied by modulation of downstream targets, leading to cell cycle arrest and apoptosis in colorectal cancer cells (Toden et al., 2015).

**FIGURE 1:**
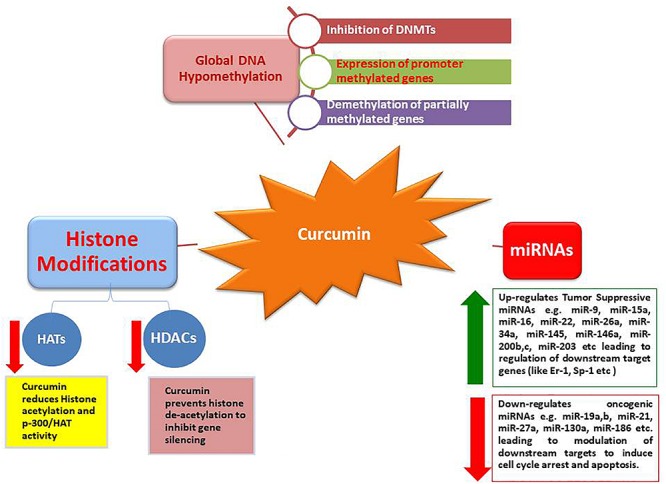
Epigenetic modulations induced by curcumin.

**FIGURE 2:**
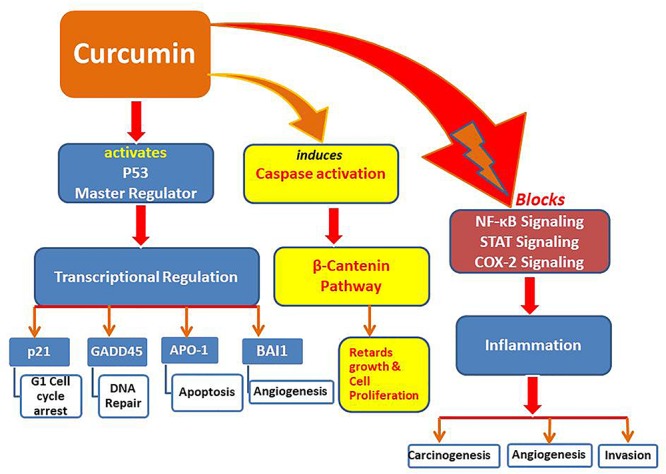
Pathways modulated by curcumin to induce tumor suppression and apoptosis.

These results provide strong evidence that bioactive compounds like curcumin could be utilized as a successful remedy against cancers, particularly when traditional therapeutics are combined with natural chemo-preventive compounds which are generally safe for humans (Li et al., 2010). Synergistic effects of epigenetic changes (de-methylation and histone acetylation/deacetylation) induced by curcumin make it the most promising anticancer molecule that can also selectively up-regulate tumor suppressive miRNAs while down-regulate oncogenic miRNAs, leading to arrest cancer progression (Figures 1, 2).

### Curcumin and Binding of DNA

The molecular foundations for various therapeutic modes of actions of curcumin are not well known due probably to the reason that so far most of the research work has mainly focused on the potent macromolecular targets of curcumin, i.e., proteins. Whereas, less attention has been paid to its potential to directly bind with DNA and/or to directly regulate epigenetic processes as an agent of DNA binding. In the year 2004, techniques of absorption spectroscopy and circular dichroism were used to demonstrate a direct communication among curcumin and both synthetic and natural duplexes of DNA. Analysis of molecular modeling estimations and spectral data inferred the DNA binding ability of curcumin. Curcumin is also a capable molecular probe to investigate naturally significant conformational polymorphisms induced by cations and pH within DNA/RNA (Zsila et al., 2004). Therefore, it could be recognized as a novel phenolic minor groove-binding factor with potent anticancer ability and therapeutic potential. Similarly, by using the techniques of UV analysis and Fourier transform infrared (FTIR), it was found that curcumin has the ability to bind with minor and major grooves of the DNA duplex, with the backbone phosphate group and also with the RNA base (Nafisi et al., 2009). Interface between these biopolymers and curcumin showed no change in conformation. However, Nafisi et al. (2009) reported that thymine O_2_ attaches curcumin to the minor groove of DNA while guanine and adenine N_7_ facilitate curcumin to bind not only to the major groove but also to the backbone phosphate group. Further, uracil O_2_ causes RNA binding whereas guanine and adenine N_7_ atoms facilitate the binding to the backbone phosphate group. Interestingly, the binding of curcumin with DNA was stronger as compared its binding to RNA.

Pentamidine is basically a diarylamidine antibiotic used for treating leishmaniasis, pneumonia, and trypanosomiasis (Fairlamb, 2003). It functions by interacting with the genome of pathogens through selective binding with the DNA’s minor groove, just like curcumin, and disrupts regular activities of the pathogenic topo-isomerases (Neidle, 2001; Bischoff and Hoffmann, 2002). Curcumin has also been recommended for the treatment of trypanosomiasis (Araujo and Leon, 2001; Saleheen et al., 2002).

### Influence of Curcumin on Transcriptional Factors

Transcriptional factors are proteins which can selectively bind to enhancer or promoter regions (possibly at histone tails) to regulate the expression of many genes. A number of transcriptional factors have been discovered and characterized with functionally diverse DNA binding and activation domains in many organisms. Many transcriptional factors possess therapeutic potential owing to their involvement in key regulatory pathways (Shishodia et al., 2007). For instance, transcription factors like NF-κB, signal transducer and transcription activator (STAT) and activator protein-1 (AP-1) regulate the expression of the genes which control cellular proliferation, survival, metastasis, invasion, transformation, programmed cell death, adhesion, and angiogenesis (Aggarwal, 2004; Aggarwal et al., 2009; Gupta et al., 2010).

Transcriptional factors which play significant roles in carcinogenesis include electrophile response element (EpRE), β-catenin, early growth response-1 (Egr-1), androgen receptor (AR), peroxisome proliferator-activated receptor-c (PPAR-c), and NF-E2-related factor 2 (Nrf2). Curcumin has been shown to inhibit NF-κB activation leading to suppression of cigarette smoke induced NF-κB-dependent cyclin D1, cyclooxygenase-2, and matrix metalloproteinase-9 expression through mediating IκBα kinase pathway in human lung carcinoma (Shishodia et al., 2003). Modulation of NF-κB (down-regulation) by curcumin was suggested to be reduced by the inhibition of IκB kinase (IKK). Curcumin also has the potential to repress active NF-κB within mantle cell lymphoma by suppressing the activity of IKK (Shishodia et al., 2005). Consequently, the levels of matrix metalloproteinase-9, cyclooxygenase-2, and cyclin D1 were down-regulated. Moreover, curcumin caused the inhibition of NF-κB pathway induced by paclitaxel within breast cancerous cells as well as the suppression of human breast cancerous cells in nude mice (Aggarwal et al., 2005).

The activator proteins play a key role in the process of tumorigenesis owing to their ability to transform cancer cells (Karin et al., 1997). The inhibition of tumorigenic factors that can activate AP-1 and JNK has been observed in response to curcumin treatment (Huang et al., 1991; Chen and Tan, 1998). Curcumin induced inhibition of AP-1 was a result of its direct interface with AP-1 DNA binding motif (Bierhaus et al., 1997). The activation of both STAT_3_ and NF-κB was inhibited by curcumin, leading to the down-regulations of genes involved in apoptosis and cellular proliferation (Mackenzie et al., 2008). Curcumin has also exhibited the inhibition of STAT_3_ phosphorylation, leading to the induction of apoptosis in cellular myeloma (Bharti et al., 2003, 2004).

It is well established that curcumin is a proteasome inhibitor that increases p53 and induces apoptosis through caspase activation (Bech-Otschir et al., 2001; Jana et al., 2004). Many studies have described curcumin mediated effects on proteasome, but the exact mechanism of proteasome inhibition has not been clearly validated. Recently, Banerjee et al. (2018) showed that curcumin is a highly effective inhibitor of dual-specificity tyrosine-regulated kinase 2 (DYRK2). They reported that curcumin disrupts 26S proteasome activity by inhibiting DYRK2 in different cancerous cells, leading to the inhibition of cell proliferation in mice. This finding opens up a new horizon for using curcumin as a potential preventive and treatment approach in proteasome-linked cancers (e.g., triple negative breast cancer and myeloma). The interaction of curcumin with a variety of cellular molecules is due to its adaptable chemical structure. Such interactions lead to diversified biological effects, including regulation of the cell cycle, growth suppression, the induction of developmental distinction, the scavenge of reactive oxygen species (ROS), chemo-prevention and the up-regulation of pro-apoptotic factors (Surh, 1999; Chauhan, 2002; Miquel et al., 2002; Saleheen et al., 2002; Taher et al., 2003; Itokawa et al., 2008).

## Other Potential Effects of Curcumin

Numerous studies have reported multiple potential effects induced by curcumin which basically stem from the interaction of curcumin with DNA, RNA, and proteins. Most important effects can be categorized as antioxidant activity, wound healing, modulation of angiogenesis and inflammatory cytokines.

### Anti-oxidant Effects of Curcumin

Oxidative stress plays significant contributing roles in many diseases such as cerebral ischemia, myocardial ischemia, shock and hemorrhage, hypoxia, cancer and the injury of neuron cells. Curcumin possesses more potent antioxidant activities than recognized antioxidants (e.g., vitamin E and C), making it a potent therapeutic agent in many inflammatory diseases (Toda et al., 1985). Curcumin is an ideal scavenger of a wide range of reactive oxygen species (Reddy and Lokesh, 1994; Unnikrishnan and Rao, 1995; Sreejayan and Rao, 1997). Moreover, curcumin had capacity for the suppression of lipid peroxidation in various animal models (Sreejayan and Rao, 1994). Curcumin induced the inhibition of lipid peroxidation, leading to the protection of renal cells (LLC-PK1) from oxidative injury (Cohly et al., 1998). It also mediated biochemical modifications induced by ischemia in the heart in a feline model (Dikshit et al., 1995).

Treatment of vascular endothelial cells with curcumin increased the expression of heme oxygenase, leading to the alleviation of oxidative damage (Motterlini et al., 2000). Curcumin has also proven its efficacy in protecting rat myocardium from myocardial ischemic injury induced by isoprenaline (Nirmala and Puvanakrishnan, 1996; Manikandan et al., 2004). These beneficial effects of curcumin were mainly associated with its ability in ROS scavenging and the inhibition of lysosomal enzymes (Nirmala et al., 1999). Treatment with curcumin also exhibited beneficial effects in kidney damage by limiting the expression of Fas-L and Fas (Jones et al., 2000).

Curcumin mediated subcellular redistribution by modulating protein kinase pathways in hypoxic rabbit hearts and exhibited the translocation of Hsp70i from the particulate to the cytosolic fraction (Rafiee et al., 2003). Supplementation of curcumin in diet has shown desirable effects in neurodegenerative disorders like Alzheimer’s disease (Calabrese et al., 2003; Yang et al., 2005). Neuroprotection by curcumin was mediated through preventing lipid peroxidation, decreasing peroxynitrite formation but increasing endogenous antioxidant enzymes within a cerebral ischemia model of rats (Thiyagarajan and Sharma, 2004). Conclusively, it has been revealed that curcumin is valuable in alleviating many ailments that originate due to oxidative stresses. Such defensive properties of curcumin are chiefly due to its antioxidant potential and thus it should be exploited to produce new therapeutic options to fight against fatal diseases.

### Improving Wound Healing by Curcumin

Repairing tissues and healing wounds are complex mechanisms which include processes like soreness, granulation and remodeling of tissues. A complicated sequence of the processes is initiated after injury, including interface between various cytokines, proteins of extra-cellular matrix (ECM), growth factors and their regulators. Previous knowledge about the potential of curcumin in injury healing prompted for the evaluation of curcumin’s effect in improving wound healing. Biopsies of wounds treated by curcumin indicated multiple infiltrating cells like fibroblasts, neutrophils, and macrophage in comparison with untreated injuries. Wound contraction was accelerated due to increased migration of myofibroblasts, fibroblasts, and macrophages within wounds treated by curcumin (Sidhu et al., 1998). Relocation of different cells serves as an effective source of growth factors needed for the maintenance of numerous natural processes of wound healing. Fibronectin (FN) and collagen expression are stimulated by transforming growth factor beta 1 (TGF-β1) along with the increase of *in vivo* development of granulation tissue during wound healing (Quaglino et al., 1990). Treatment with curcumin enhanced the expression of collagen and FN (Sidhu et al., 1998).

Further, curcumin enhanced the creation of granulation tissue, including rapid re-epithelialization, greater cell content, and neo-vascularization of wound impaired with hydrocortisone as well as diabetes (Sidhu et al., 1999) by controlling the expression of TGF-β1, its receptors and nitric oxide synthase during wound healing (Mani et al., 2002). Modulation of NF-κB activity by curcumin exerts advantageous effects by increasing the regeneration of muscles soon after trauma (Thaloor et al., 1999). Many reports suggest an antioxidant role of curcumin in wound healing by indicating its ability to prevent damage resulting from hydrogen peroxide in fibroblasts and keratinocytes in humans (Phan et al., 2001). Similarly, treatment of collagen matrix with curcumin exhibited rapid wound repair, better cell proliferation, and effective foraging of free radicals when compared with collagen treated and control rats (Gopinath et al., 2004). Pre-treatment with curcumin improved the formation of nucleotides, collagen, hexosamine, and nitrite. Histological investigation of injury biopsies revealed a better deposition of collagen and increased vascular and fibroblast levels, indicating the potential role of curcumin in improving radiation-induced delay in wound healing (Jagetia and Rajanikant, 2005).

Anti-oxidative potential of curcumin also makes it a promising anti-ulcer agent owing to its defensive ability against lipid peroxidation, depletion of glutathione and oxidation of proteins. Curcumin not only accelerated wound repair but also protected against gastric ulcer by improvement in MMP-2 activity and reduction of MMP-9 activity (Swarnakar et al., 2005). These findings evidently recommended the effectiveness of curcumin in wound healing, improved the control with respect to the formation of granulation tissue along with the stimulation of growth factors. It is evident that curcumin supplements wound healing at various levels. Recently, nano-curcumin showed mobilization of fibroblasts at wound site by activating the Wnt signaling pathway partly mediated through Dickkopf-related protein-1. Moreover, it also exhibited persistent inhibition of the inflammatory response through decreasing monocyte chemoattractant protein-1 (Dai et al., 2017).

### Angiogenesis Modulation by Curcumin

Angiogenesis is of vital importance in many physiological activities, including reproductive process, embryo development, bone repair, and wound healing. Contrarily, irregular angiogenesis has been found to be pathological and linked with the growth of tumors, retinopathy of diabetes, hemangiomas, and rheumatoid arthritis. The role of angiogenesis regarding to the growth of primary tumors along with their metastasis to remote organs is well established (Folkman, 1995). Studies have revealed the potent anti-angiogenic ability of curcumin (Thaloor et al., 1998). Moreover, curcumin has also shown to inhibit corneal neo-vascularization in the mouse cornea (Arbiser et al., 1998). Such therapeutic effectiveness in the cornea has also been suggested after the provision of dietary curcuminoids in mice (Mohan et al., 2000). Reports have shown that certain analogs of curcumin may exhibit angiostatic abilities (Ahn et al., 2002; Shim et al., 2002). Similar consequences of curcumin analogs were also documented for genes of MMP-9 and VEGF (Hahm et al., 2004). It is well recognized that MMP genes and related particular suppressors have a key role in regulating the re-organization of matrix and initiating angiogenesis (Schnaper et al., 1993). The modulation of MMPs (accountable for the reduction of angiogenic activities) has been shown by curcumin and its analogs (Thaloor et al., 1998; Kim et al., 2002; Hahm et al., 2004).

### Mediation of Inflammatory Cytokines

Curcumin has shown the repression of downstream pro-neoplastic and pro-inflammatory mediators, including reduced expression of IL-8 and IL-6 as a reaction to acidic contact in human oesophageal epithelial cell lines (Rafiee et al., 2009). It also decreased impulsive expression of IL-8 and IL-6 within four varied squamous carcinoma cell lines of the head and neck. These results revealed curcumin-induced inhibition of intermediary signaling pathways like NF-κB. Furthermore, curcumin also suppresses the production of interleukin 8 and 6 in neck and head cancerous cells by inhibiting Iκ β kinase in humans (Cohen et al., 2009).

Pretreatment by curcumin restored hepatic cytokines, including IL-1α and β, IL-2, IL-6, and IL-10 to normal intensities after injuries. Moreover, NF-κB and AP-1 were activated differentially at 2 and 24 h after hemorrhage. Liver injury was reduced by serum aspartate transaminase in animals pretreated by curcumin which were suffering from severe hemorrhage. Such consequences showed that the defense provided by curcumin against resuscitation/hemorrhage damage may be due to the inactivation of transcriptional factors responsible for cytokine regulation (Gaddipati et al., 2003). Under different experimental conditions, curcumin is recognized as a capable anti-inflammatory factor (Pari et al., 2008). Curcumin mediated the expression of ultraviolet or TNF-α-induced inflammatory cytokines in human cells (Jiang et al., 2009; Reuter et al., 2009; Wang et al., 2009). All these molecular modulations induced by curcumin with vital health benefits could be exploited in alternative therapeutics to manage severe diseases in humans (Figure 2).

## Conclusion

Evidence provided by diversified experiments conducted in recent years support the argument that dietary phytochemicals like curcumin have significant strengths as epigenetic modulators. Epigenetic alterations may be modulated through nutritional, pharmacological and environmental interference. This feature has stimulated the insight for development of therapeutic approaches focusing on different epigenetic factors, including HAT, DNMTs, miRNAs, and HDAC, by addition of dietary polyphenols like curcumin. Curcumin is a versatile molecule having adaptable structure with diverse biological functions. Curcumin is a potent proteasome inhibitor that increases p53 level and induces apoptosis by mitochondrial caspase activation. Curcumin also disrupts 26S proteasome activity by inhibiting DYRK2 in different cancerous cells, resulting in the inhibition of cell proliferation. However, further research work is required to explore the full epigenetic potential of curcumin for preventing and curing lethal diseases like cancers.

## Author Contributions

FH conceived the idea and drafted the outline. MAA, AJ, and AN collected the literature and wrote the different sections of manuscript. FH, MSR, and MSK edited and revised the manuscript. FH and CY made final changes, edited the manuscript, and finalized table and figures.

## Conflict of Interest Statement

The authors declare that the research was conducted in the absence of any commercial or financial relationships that could be construed as a potential conflict of interest.
